# Stereoacuity among Undergraduate Medical and Nursing Students at a Tertiary Care Hospital: A Descriptive Cross-sectional Study

**DOI:** 10.31729/jnma.7053

**Published:** 2022-01-31

**Authors:** Pragati Gautam Adhikari, Sangam Shah, Nikita Bhatta, Prince Mandal, Basanta Sharma Paudel, Apil Pokhrel, Bipin Koirala, Chiranjiwi Prasad Shah

**Affiliations:** 1 Department of Ophthalmology, Maharajgunj Medical Campus, Institute of Medicine, Tribhuvan University, Maharajgunj, Kathmandu, Nepal; 2 Maharajgunj Medical Campus, Institute of Medicine, Tribhuvan University, Maharajgunj, Kathmandu, Nepal; 3 Maharajgunj Nursing Campus, Institute of Medicine, Tribhuvan University, Maharajgunj, Kathmandu, Nepal; 4 Tribhuvan University Teaching Hospital, Maharajgunj, Kathmandu, Nepal

**Keywords:** *binocular vision*, *depth perception*, *stereopsis*

## Abstract

**Introduction:**

Stereopsis is defined as the ability to perceive object depth. It is measured in seconds of arc. Reduced stereoacuity impinges one's academic as well as a professional performance. Hence in this study, we aim to find out the mean stereoacuity among the undergraduate medical and nursing students using the Titmus fly test.

**Methods:**

A descriptive cross-sectional study was conducted among undergraduate students at a medical college of Nepal from April 2021 to July 2021. Ethical approval was obtained from the Institutional review committee (Registration number: 487 (6-11) E2 077/078). Simple random sampling method was used. Data were collected from undergraduate medical and nursing students while the post-graduate students were excluded from the study. Overall, 80 students were included in the study. Titmus fly test was used to assess the stereopsis. Statistical Package for Social Sciences version 21 and Microsoft Excel was used for data analysis. Point estimate at 95% confidence interval was calculated along with mean, standard deviation, frequency, and proportion.

**Results:**

The mean stereoacuity was 62.63±46.56 (range 40-800) sec of arc (52.77-72.49 at 90% Confidence Interval). A total of 80 participants in our study among which 50 (62.5%) were male and 30 (37.5%)were female. About 41 (51.2%) of the study participants had normal, borderline (>40 and ≤ 120) stereopsis was seen in 35 (43.8%) of the study participants while only 4 (5%) had reduced stereopsis (≥120 sec of arc).

**Conclusions:**

This study showed that the mean stereoacuity among the undergraduate medical and nursing students was in subnormal range which was similar to other studies.

## INTRODUCTION

Stereopsis is defined as the ability to perceive object depth and happens due to fusion of two slightly different images produced by stimulating two separate retinae on the Panum's area to form binocular single vision.^1,2^ There is a small horizontal disparity in the fused retinal images. The small horizontal disparity creates depth perception. The horizontal disparity is expressed in terms of angle subtended at the nodal point of eye.^2,3^

The least horizontal difference is referred to as the threshold disparity and is measured in seconds of arc. A stereoacuity of fewer than 40 seconds is considered normal.^4,5^ It is influenced by reduced visual acuity, presbyopia, amblyopia, strabismus, and other various visual defects. There is importance of stereoacuity in medical practice^6^ and reduced stereoacuity impinges one's academic as well as professional performance.

Hence, we aimed to find out the mean stereoacuity among undergraduate medical and nursing students using Titmus fly test.

## METHODS

A descriptive cross-sectional study was employed to find out the mean stereoacuity among the undergraduate medical and nursing students of the Institute of Medicine using theTitmus fly test . This study was conducted on two campuses under the Institute of Medicine that is Maharajgunj Medical Campus (MMC) and Maharajgunj Nursing Campus (MNC). Ethical approval was obtained from the research ethics committee of the Institutional Review Committee (IRC) of the Institute of Medicine (IOM) [Ref: 487 (6-11) E2 077/078]. Official letters of cooperation from IRC were written to respective study districts and companies. Informed consent was obtained from all study subjects to allow the use of anonymous personal and clinical data in research. Confidentiality of the information was maintained thoroughly by de-identification.

The study was conducted from April 2021 to July 2021. The study population was a group of 80 medical students of MMC and MNC from different faculties. Undergraduate medical and nursing students were included while the post-graduate students were excluded from the study. The students who denied consent were not included in the study. The researcher intends to perform the study among students taking into account their age, gender, caste, religion, address, and stereoacuity by Titmus test. The students were chosen using simple random sampling (lottery method), considering the sample's representativeness and logistical issues.

The sample size was calculated using the formula as given below:

n = Z^2^ × σ / e^2^

  = (1.96)^2^ × 2.09^2^/ (0.5)^2^

  = 68

Where,

n= minimum required sample size
Z= 1.96 at 95% Confidence Interval (CI)
σ= standard deviation calculated considering upper and lower margin as 600secs and 98secs at 95% Confidence Interval respectively (educated guess)
e= lower margin of error taken as 0.5

Taking the finite population, i.e., total students of MMC and MNC (N)= 1200

Adjusted sample size= n/[1+{(n-1)/N]}]

= 68/[1+{(68-1)/1200}]

= 65

Thus, the minimum number of the sample size required was calculated as 65. By adding 10% as a non-response rate, the minimum sample size was 72 and a sample size of 80 was taken.

Using simple random technique, 80 medical students from two campuses were randomly picked without replacement from a full list of respondents acquired from students.

Titmus fly test uses crossed Polaroid filters to present slightly different aspects of the same object to each eye. The test comprises three sections of which the housefly shows large disparities, circle patterns that consist of patterns containing four circles. One of each four circles is graded. The third is animal, altogether there are 3 rows of animals, one animal in each row has a graded disparity.

Students' age, gender, caste, religion, address, history of strabismus, family history of stereopsis, and steroacquity by titmus test were the variables for this study. The data were collected in the proforma. Personal interviews and examinations were made to acquire the relevant information from the students. Tables, charts, figures, and statistical tools were used to present the study's findings. Statistical Package for Social Sciences version 21 and Microsoft Excel 16 were used for data entry and analysis. Point estimate at 95% confidence interval was calculated along with mean, standard deviation, frequency, and proportion.

## RESULTS

Among 80 students the mean stereoacuity is 62.63±46.562 sec (range 40 - 800) sec of arc (52.77-72.49 at 90% Confidence Interval). Forty-one (51.2%) of the study participants had normal stereopsis. Borderline (>40 and ≤ 120) stereopsis was seen in 35 (43.8%) of the study participants. While only 4 (5%) had reduced stereopsis (≥120 sec of arc) (Table 1).

**Table 1:** Distribution of stereopsis (n = 80).

	n (%)
Normal (up to 40 sec of arc)	41 (51.2)
Borderline (>40 and ≤ 120)	35 (43.8)
Reduced (≥120)	4 (5.0)

The stereoacuity was found to be 64±46.562 sec of arc in males which improved comparatively in females with stereoacuity 60.33±30.904 sec of arc. After undergoing further analysis, a larger angle of binocular disparity (lower stereoacuity) was found to be associated with participants having refractive errors. The distribution of refractive errors, having strabismus, is shown below (Table 2).

**Table 2:** Distributions of populations with their means (n = 80).

		Stereoacuity (sec of arc) Mean±SD
Sex	Male	64.00±57.286
	Female	60.33±30.904
Refractive error	Yes	102.22±95.182
	No	51.13±23.547
Strabismus history	Yes	420.00±417.401
	No	53.46±23.122
Family history of stereopsis	Yes	220.00±204.962
	No	52.13±22.499

The number of hours spent using digital devices and degrees of stereoacuity are shown below (Table 3).

**Table 3:** Distribution of stereopsis and working hours (n = 80).

		Normal	Borderline	Reduced
working	≤ 4hrs	22	18	2
hours	> 4hrs	19	17	2
Total		41	35	4

Out of 80 participants, refractive errors were evident in 18 (22.5%) and 62 (77.5%) had normal visual acuity. All the study population with refractive errors were wearing glasses. Two (2.5%) participants had a history of strabismus while 78 (97.5%) participants didn't have strabismus. About 5 (6.3%) participants had a family history of strabismus (Table 4).

**Table 4:** Eye-related issues among study participants (n = 80)

	n(%)
**Strabismus history**	
Yes	2 (2.5)
No	78 (97.5)
**Wearing glass**	
Yes	18 (22.5)
No	62 (77.5)
**Family history of strabismus**	
Yes	5 (6.3)
No	75 (93.8)
**Comorbidities**	
Hypertension	1 (1.3)
Diabetes	1 (1.3)
pulmonary TB	1 (1.3)
No comorbidities	77 (96.3)

In a total of 80 participants in our study, 50 (62.5%) were male and 30 (37.5%) were female. The mean age of the study participants was 22.34±1.691 years (range: 20-29 years). About 54 (67.5%) of participants were Brahmin, 7 (8.8%) were Chettri, and 12 (15%) were Madhesi while the lowest proportion was of Janajati 6 (7.5%). Similarly, 72 (90%) of the student were Hindu, 4 (5%) were Christian, 3 (3.75%) were Buddhist, and 1 (0.25%) was Muslim (Table 5).

**Table 5:** Socio-demographic participants (n = 80).

	n (%)
**Sex**	
Male	50 (62.5)
Female	30 (37.5)
**Ethnicity**	
Brahmin	54 (67.5)
Chetri	7 (8.8)
Janajati	6 (7.5)
Madhesi	12 (15.0)
Others	1 (1.3)
**Religion**	
Hindu	72 (90)
Christian	4 (5)
Buddhist	3 (3.75)
Muslim	1 (0.25)
	Mean±SD
Age (in years)	22.34±1.691

## DISCUSSION

Among 80 students, 62.63±86.562 was the mean stereoacuity (Confidence Interval= 43.662 - 81.598). This is the first study in the Tribhuvan University population for the assessment of stereopsis in adults. The mean adult near stereo-acuity of participants with normal binocular vision is 20 seconds of arc, with a standard deviation of 10 seconds of arc, according to Ogle.^7^ 95% of the normal group in his study had stereoacuity thresholds of 40 seconds of arc. However, in our study population, 51.2% had normal stereo acuity of 40 arc seconds, and 43.8% had near-normal stereo acuity of up to 120 arc seconds. The higher stereo acuity levels were enjoyed by people before 60 years of age in various studies.^5,8^ In the current study, however, this number is substantially lower.

Stereopsis is connected with the quality of life in many aspects of human life. Stereopsis is the only direct measurement of depth perception even though various other monocular cues can provide an indirect measurement. The importance of stereopsis in professions requiring a high level of visual skill remains debatable. Stereoacuity is necessary for all jobs. There is evidence of the importance of stereopsis for the medical practitioner as well whose job is related to small object manipulation.^9-11^ The some countries' stereopsis is one of the criteria for selection in residency training.^12^ In a study done by Suleman, et al. it was found that medical students with a depth-perception defect performed worse on a basic laparoscopic task.^13^ The current study tries to explore the stereoacuity in future medical practitioners. The majority of them had normal stereoacuity and they presumably can have better surgical or other fine skills based on this information.

3D technology harms the visual system, causing three unnatural distortions. The fusion is poor along with stereopsis distortion and vergence-accommodation discrepancy. These are the factors that cause stereoacuity to deteriorate.^14^ Furthermore, looming, motion parallax, and the kinetic depth effect, as well as pictorial depth cues such as occlusion, perspective, texture gradients, relative size, and height in the visual field, shadow, brightness, and aerial perspective, all influence stereopsis.^15,16^ As a result of these characteristics, the number of hours spent using digital devices may affect degrees of stereoacuity however in our study there was not much difference in stereoacuity among those spending more hours with digital devices than among those spending fewer hours. Similar findings were found in a study done among college students.^16^

The level of stereoacuity affects fine motor skills in children. Ocular diseases such as ametropia, aniseikonia, amblyopia, strabismus, nystagmus, aphakia, and monovision and monofixation syndrome can all impair the development of stereopsis.^17^ In our study only 2% of study participants had a history of strabismus, with 6% having a family history of strabismus. This might explain the relatively low 4% level of reduced stereopsis in our study. Poor best corrected visual acuity had a significant influence on stereopsis as shown by several studies.^18-20^ In our study too refractive errors were evident in 22.5% of participants among them stereopsis was low compared to participants with normal uncorrected visual acuity. Though all of the study participants wore glasses, their refractive error might not be fully corrected. More so anisometropia may also affect stereopsis.^21^ The precise mechanism by which anisometropia causes a decrease in stereoacuity is not clear. This aspect however was not fully explored in our study to put forward conclusive remarks on this part.

Though age might affect stereopsis.^8,22,23^ In the current study females were found to have relatively better stereoacuity than males. Few studies have found differences in stereoacuity among gender but it was not statistically significant. In a similar study done among students, females were found to have better stereopsis than males.^24^

There are some limitations to our study, such as the fact that we only asked a single question about the amount of time spent using digital devices. We also did not take into account the types of devices used and refractive errors among the medical students. The random-dot test is considered superior to assess binocular vision instead of the titmus fly test.^25^ Our study had a small sample size thus, limiting the findings. Also, being a descriptive cross-sectional study, our findings cannot make an association among the variables or prove causality. A future study could be conducted by utilizing a specific period of digital technology that can be continuously maintained to measure pre- and post-stereopsis. To light further insight on the stereopsis and its significance, a large study sample must be chosen.

## CONCLUSIONS

This study showed that the mean stereoacuity among the undergraduate medical and nursing students was in a subnormal range which was similar to other studies. It may be a major reason for them not performing well in medical school and, as a result, harm their careers. So, routine screening of stereo acuity levels in medical students should be performed to identify this defect in binocular vision, and when possible corrective measures should be sought as simple as correcting refractive errors.
